# Serotonin promotes calcium accumulation and inhibits lipid accumulation in cultured goat mammary epithelial cells through HTR2A

**DOI:** 10.5713/ab.24.0792

**Published:** 2025-04-04

**Authors:** ZhiFei Zhang, BinHan Li, YingFei Wang, Tumaresi Abuduwufuer, Kang Hu, HuiLing Zheng

**Affiliations:** 1 Key Laboratory of Animal Genetics, Breeding and Reproduction of Shaanxi Province, College of Animal Science and Technology, Northwest A&F University, Xianyang, China; 2 Key Laboratory of Animal Nutrition and Feed Science in South Chian, Ministry of Agriculture and Rural Affairs; Guangdong Provincial Key Laboratory of Animal Breeding and Nutrition; Institute of Animal Science, Guangdong Academy of Agricultural Sciences. Guangzhou, China; 3 Key Laboratory of Livestock Biology, Northwest A&F University, Xianyang, China

**Keywords:** Calcium, Goat, Lipid Synthesis, Mammary Epithelial Cells, Serotonin

## Abstract

**Objective:**

Fatty acids and calcium are both important nutrients in goat milk. Investigating the upstream molecular regulatory mechanisms that control the synthesis of milk fat and milk calcium in the mammary gland can help improve the quality of milk at its source. The objective of this study was to investigate the effects and regulatory pathways of serotonin (5-hydroxytryptamine, 5-HT) and its receptors on lipid synthesis and calcium ion levels in goat mammary epithelial cells (GMECs).

**Methods:**

GMECs isolated from live goats were treated with serotonin, overexpression of *Serotonin receptor* 2A (HTR2A), Sarpogrelate ([SAR] the specific antagonist of HTR2A), or a combination of these agents. The expression of genes related to *de novo* lipid synthesis in GMECs were detected using the Quantitative Real-Time polymerase chain reaction, the content of lipid droplets was detected using the BODIPY assay, and the calcium content was detected using the calcium chelating probe Fluo-3AM assay.

**Results:**

5-HT dose-dependently promotes the activity of GEMCs, significantly inhibits the mRNA expression of key genes involved in de novo lipid synthesis such as *ACC*, *FASN*, *SREBP1*, *SCD1* and *ELOVL6* at a concentration of 100 μM, reduces triglyceride and total cholesterol content, suppresses lipid droplet accumulation in cells, and simultaneously promotes calcium accumulation in cells. Furthermore, overexpression of *HTR2A* in GMECs also induces an increase in cellular calcium levels and inhibits lipid synthesis and accumulation in cells. However, treatment of cells with SAR, the specific antagonist of HTR2A, significantly increases the levels of triglycerides, total cholesterol, and lipid droplet accumulation in cells.

**Conclusion:**

5-HT inhibits lipid synthesis in GMECs while promoting an increase in cellular calcium levels, and this effect is mediated by the HTR2A receptor. Furthermorey, antagonists targeting HTR2A can reverse the inhibition of lipid synthesis and accumulation in cells.

## INTRODUCTION

Fatty acids and calcium are important nutritional components in milk, especially goat milk. They not only create the unique flavor of dairy products but also help to provide essential nutrition for dairy products and promote the development of bone and nerves [1,2]. While low-fat, high-calcium milk is increasingly favored by consumers, there remains a scarcity of research on the production of naturally low-fat, high-calcium milk. Mammary epithelial cells, serving as the primary source of lipids and calcium in dairy products, play a crucial role in processes such as lipid accumulation, metabolism, and calcium ion transport, all of which are intricately linked to the functionality of mammary glands [3,4]. Therefore, addressing the aforementioned research gap, it is imperative to investigate the upstream regulatory pathways governing lipid accumulation and calcium metabolism in mammary epithelial cells.

Serotonin, a monoamine discovered 70 years ago, is synthesized by converting tryptophan into 5-hydroxytryptophan by tryptophan hydroxylase (TPH1) and then by 5-hydroxytryptophan decarboxylase [5]. During the last 3 decades, 5-hydroxytryptamine (5-HT) functions in the peripheral system have greatly emerged with the cloning of at least 15 kinds of 5-HT receptors [6,7]. Through its effects on cellular junctions, cell proliferation, apoptosis, and survival, 5-HT is of great significance to the development and degeneration of mammary glands [8]. It is now well established that 5-HT functions as a “local mammary homeostasis regulator” for its roles in the activation of secretory and integration of calcium mobilization [9,10]. Our previous studies have confirmed that administering 5-hydroxytryptophan to peripartum dairy goats can elevate both circulating calcium levels and calcium ion concentrations in goat milk [11]. Treatment of goat mammary epithelial cells (GMECs) with 5-HThas been shown to suppress cell apoptosis. Furthermore, we utilized CRISPR-Cas9 technology to specifically knock out *TPH1* in GMECs, resulting in a significant reduction in cellular calcium content under this condition [12]. While studies in cows suggest that 5-HTmay impact lipid metabolism or energy metabolism in mammary tissue [13], few studies have simultaneously investigated the influence of 5-HT on lipid accumulation and calcium ion content in the mammary gland of dairy goats.

The biological realm currently recognizes fourteen different 5-HT receptors, a fact that not only underscores the complexity of 5-HT function but also provides researchers with clues to decipher molecular pathways regulating specific functions of 5-HT. In liver and adipose tissue, serotonin receptor 2A (HTR2A) has been reported to be associated with cellular lipid metabolism [14,15]. Therefore, the present study focused on mammary epithelial cells’ lipid synthesis and calcium ion content, investigating the molecular mechanisms by which 5-HT and HTR2A concurrently regulate cellular lipid synthesis and calcium accumulation. Additionally, the study validated the effects of specific antagonists targeting this receptor, aiming to provide a theoretical basis for precise regulation of lipid and calcium metabolism in GMECs.

## MATERIALS AND METHODS

### Animal care

All the experimental procedures were approved by the Institutional Animal Care and Use Committee of the Northwest A&F University, Yang Ling, Shaanxi, China (agreement No: 15-516).

### Isolation and culture of primary goat mammary epithelial cells

The protocol of GMECs’ isolation, purification and the authenticated procedure have been described in detail in previous studies [16]. In brief, GMECs were isolated from mammary gland biopsies of 3 goats at the peak lactation stage. Under sterile conditions, mammary gland tissue sections were dissected and washed with D-Hank’s solution. The granular acinar tissue was cut into small pieces (1 to 2 mm) and then cultured with complete medium until cells separated from the tissue. The isolated cells were subjected to immunofluorescence staining for KRT1 protein, a marker of epithelial cells, to confirm their identity as mammary epithelial cells (Supplement 1). The GMECs were kept in complete culture DMEM/F12 (D6570; Solarbio, Beijing, China) medium containing 10% fetal bovine serum (Gibco, Gaithersburg, MD, USA), 100 U/mL penicillin/streptomycin (080092569; Harbin Pharmaceutical Group, Harbin, China), 5 μg/mL bovine insulin (16634; Sigma Aldrich, St. Louis, MO, USA), 10 ng/mL epidermal growth factor (PHG0311; Sigma Aldrich) and 5 ng/mL hydrocortisone (H0888; Sigma Aldrich) in 5% CO_2_ at 37°C. To promote lactogenesis, the cells were cultured in a lactogenic medium which was prepared as the complete medium with prolactin (HY-P70745, 2 μg/mL, MedChemExpress, Monmouth Junction, NJ, USA) for 48 h before the following experiments.

### Preparation of drugs and treatments

Serotonin HCL (5-HT, 153-98-0; Selleck, Shanghai, China) and Sarpogrelate hydrochloride (MCI-9042, SAR, a selective HTR2A antagonist, 135159-51-2, Selleck, Shanghai, China), were dissolved in dimethyl sulfoxide (DMSO), and further dilutions were made in complete medium. Cells well were treated with DMSO or 5-HT (50, 100, 150 μM) or SAR (30 μM) either individually or in combination. Then, cells were harvested at 48 h for subsequent assays.

### Construction of HTR2A eukaryotic expression vector

The primers for the *HTR2A* gene were designed based on the complete coding sequence region of Capra hircus *HTR2A* gene, which was previously published in GenBank (NXM_005687443.3, https://www.ncbi.nlm.nih.gov/nucleotide/, accessed on 14 June 2024). The 5′ and 3′ ends of the primer were modified to include *BamH* I and *Not* I restriction enzyme cutting sites, respectively. Tsingke Biotech Co., Ltd. (Beijing, China), synthesized the primers with the following sequences: 5′-ACATACGCCAGCCTCACT-3′ and antisense 5′-TTTCT CCAGTCTCCCAGT-3′. Total RNA was extracted from tissue and cells using the TRIzol reagent (Tiangen Biotechnology Co., Ltd.), followed by reverse transcription into cDNA with the cDNA synthesis kit (TaKaRa Biotechnology Co., Ltd., Beijing, China). After the amplification of the cDNA, the nucleotide of HTR2A was amplified and subcloned into the shuttle vector pMD19-T on ice. The recombinant plasmid pMD19-T-*HTR2A* was transformed into *Escherichia coli* DH5α competent cells. The suspended cells were coated on Luria-Bertani (LB) solid medium. Several colonies were selected and inoculated into LB liquid medium. Plasmids were extracted using a plasmid extraction kit (Tiangen Biotechnology Co., Ltd.) without endotoxin. Homologous recombination occurred between the shuttle vector and backbone vector, resulting in the positive recombinants of plasmid (pcDNA3.1-HTR2A) identified by kanamycin resistance and digestion with restriction endonucleases *BamH* I and *Not* I.

### Real-time analysis of cell activity

The protocol of real-time analysis of cell activity (RTCA) have been described in detail in previous studies [17]. In brief, 50 μL of medium was added to each well of the E-Plate and the E-Plate was placed into RTCA (Real Time Cellular Analysis iCELLigence, ACEA Biosciences, San Diego, CA, USA) within 10 minutes for background impedance value detection. GMECs were seeded into wells (1×10^4^ cells/well) of the E-Plate of RTCA. Thereafter, the E-Plate was mounted on RTCA and placed in a 37°C, 5% CO_2_ incubator. The impedance value was measured every 15 minutes throughout the whole process until the end of the experiment.

### Lipid droplet staining

The GMECs were washed three times with phosphate-buffered saline (PBS). According to the manufacturer’s instructions, the lipid droplets in GMECs were stained BODIPY (D3921; Invitrogen Corporation, Waltham, MA, USA). DAPI (2 ng/μL) was used to label the cell nuclei. After 35 minutes, the cells were washed three times and examined under the microscope.

### Triglycerides/total cholesterol assay

Cells were washed three times with cold PBS buffer, and the cell cholesterol and triglycerides (TAG) were quantified by Glycerol-3-phosphate oxidase-Trinder triacylglycerol assay kit (E1013; Applypen Technologies Inc., Beijing, China) as described previously [18]. Quantification was performed with a Biotek microplate reader (Winooski, VT, USA) at 550 nm absorbance. Total protein was determined by a bicinchoninic acid (BCA) protein assay kit (Thermo Fisher Scientific, Waltham, MA, USA). Cellular TAG quantifications were quantified by normalization to total protein.

### Calcium staining

After incubation for 24 hours, the GMECs were loaded with the fluorescent calcium indicator Fluo-3, AM (Solarbio). Fluo-3, AM was dissolved in anhydrous DMSO to prepare a 2 mM storage solution. An equal volume of 20% pluronic F127 solution (Solarbio) was added to the fluo-3, AM/DMSO solution. 4 μm fluo-3, AM working solution was prepared by diluting with Hanks balanced salt solution ([HBSS] Solarbio). Fluo-3, AM working solution was then added to the cells. After incubation at 37°C for 20 minutes, five times the volume of HBSS containing 1% fetal bovine serum was added to GMECs for 40 minutes. The cells were washed 3 times and then resuscitated with HEPES buffer saline (Solarbio) to make 1×10^5^ cells/ml solution. Cells were cultured for 10 minutes, and detected using Fluo-3, AM (4 μM, Solarbio) by a laser scanning confocal microscope (Becton Dickinson, Inc., Franklin Lakes, NJ, USA). The fluorescence intensity was measured by ImageJ software. The intracellular concentrations of total calcium were also determined by using a quantitative colorimetric calcium assay kit (Nanjing Jiancheng Bioengineering Institute, Nanjing, China).

### RNA extraction and quantitative real-time polymerase chain reaction

The GMECs at a density of 3×10^5^ cells per well in 12-well plates were harvested for RNA extraction after treatments. Total RNA was extracted from tumor cells using RNAiso Plus (9109; Takara, Shiga, Japan). The quality of RNA was detected by a NanoDrop2000 spectrophotometer (Thermo Scientific), and the optical density 260/280 ratio was 1.9–2.1:1. According to the manufacturer’s instructions, 1 μg of total RNA was reversely transcribed using PrimeScript RT Reagent kit (RR047A, Perfect Real Time, Takara). Subsequently, real-time quantitative polymerase chain reaction (RT-qPCR) reactions were performed according to the manufacturer’s instructions using SYBR green (RR820A, TB Green II, Perfect Real Time; Takara) in a CFX-96 Real-Time PCR Detection System (Bio-Rad Laboratories Inc., Hercules, CA, USA). The thermal cycle settings were as follows: 95°C for 15 s, then 40 cycles of 95°C for 5 s and 60°C for 30 s. A negative RT sample and water control were run on all plates. Ubiquitously expressed transcript, ribosomal protein S9 (RPS9), and mitochondrial ribosomal protein L39 (MRPL39) were used as reference genes. Relative mRNA levels were determined using the 2^−ΔΔCt^ method. Primers were listed in Supplement 2. The amplification efficiency of the primers was confirmed to be in the range of 95% and 105%, and the specificity of the primers was evaluated by the presence of a single temperature dissociation peak.

### Western blot

Cells were lysed in RIPA buffer (Thermal Scientific) containing a protease inhibitor cocktail (Roche, Basel, Swiss). Protein concentrations were determined by using a BCA Protein Assay Kit (Thermal Scientific). Proeins (20 μg/sample/lane) were electrophoresed on 8% sodium dodecyl sulfate polyacrylamide gel electrophoresis (SDS-PAGE). Proteins were transferred to a polyvinylidene fluoride membrane. The membrane was blotted with primary antibodies for detection of specific proteins as described previously [19]. Antibodies used included: HTR2A (bs-12049R, Bioss, Woburn, MA, USA, 1:1000), AMPK (ab131512; Abcam, Cambridge, UK, 1:2000), p-AMPK (ab133448; Abcam, 1:2000), CaMKKβ (bs-6253R; Bioss, 1:1000), p-CaMKKβ (12818S; CST, Boston, MA, USA, 1:2000). Mouse monoclonal β-actin antibody (CW0096, CW Biotech, Beijing, China, 1:1000) was probed as the loading control.

### Statistical analysis

Statistical analyses were carried out using the SPSS 20.0 statistics software package. All the data were presented as mean± standard error of the mean of three independent experiments. Comparisons between two groups were analyzed by Student’s t test for unpaired samples. A one-way analysis of variance was used to determine significant differences of multi-groups. Differences were considered significant at *p<0.05.

## RESULTS

### 5-HT dose-dependently increases the cell activity of goat mammary epithelial cells

**Figure 1:**
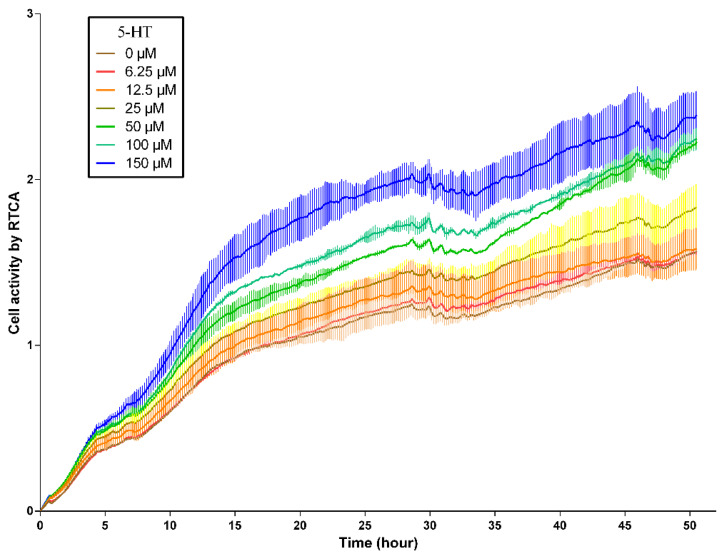
5-HT dose-dependently increases the cell activity of GMECs. The effects of 7 different concentration (shown by lines with different colors in the figure) of 5-HT within 48 hours on the activity of GMECs by RTCA assays. n = 3, Data are shown as mean±SEM. RTCA, real-time analysis of cell activity; GMECs, goat mammary epithelial cells; 5-HT, 5-hydroxytryptamine; SEM, standard error of the mean.

Firstly, to determine the optimal treatment concentration, we investigated the effects of seven different concentration of 5-HT within 48 hours on the activity of GMECs. The results demonstrated that the real-time activity of GMECs increased with the elevation of 5-HT concentration (0 to 150 μM; Figure 1).

### 5-HT inhibits lipid droplet accumulation and promotes calcium elevation in goat mammary epithelial cells

**Figure 2:**
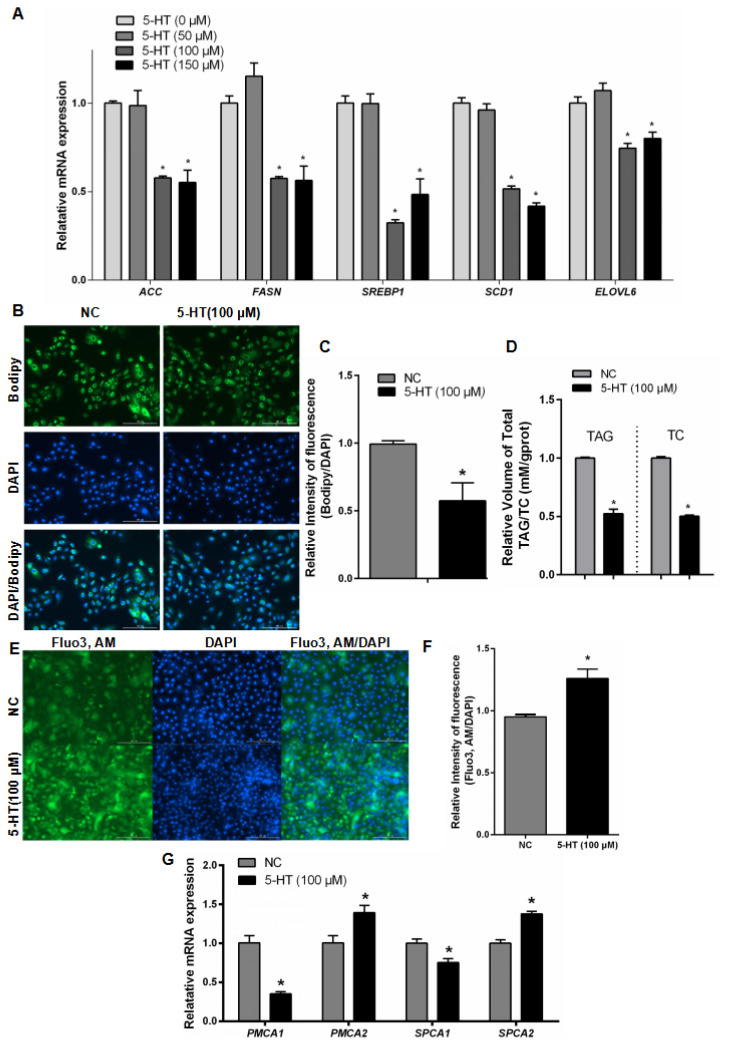
5-HT inhibits lipid droplet accumulation and promotes calcium elevation in GMECs. (A) RT-qPCR analysis of *ACC*, *FASN*, *SREBP1*, *SCD1* and *ELOVL6* expression in GMECs. (B,C) Bodipy staining and quantitative analysis of lipid droplets in GEMCs. (D) TAG/TC analysis of concentrations of total TAG and TC in GMECs. (E,F) Fluo-3 staining and quantitative analysis of intracellular calcium in GMECs. (G) RT-PCR analysis of *PMCA1*, *PMCA2*, *SPCA1* and *SPCA2* in GMECs. GMECs treated with 5-HT at 0, 50, 100, or 150 μM concentrations. Scale bar, 200 μm. Error bars indicate means±SEM, * p≤0.05. 5-HT, 5-hydroxytryptamine; NC, negative control; DAPI, 4′,6-diamidino-2-phenylindole; TAG, triglycerides; TC, total cholesterol; GMECs, goat mammary epithelial cells; RT-qPCR, real-time quantitative polymerase chain reaction; SEM, standard error of the mean.

Subsequently, GMECs were treated with three concentrations of 5-HT (0, 50, 100, 150 μM). The results showed that at concentrations of 100 μM or 150 μM, the mRNA expression levels of lipid synthesis-related genes *Acetyl-CoA Carboxylase* (*ACC*), *Fatty Acid Synthase* (*FASN*), *SREBP*, *SCD1*, and *ELOVL6* were significantly decreased (Figure 2A). Staining of lipid droplets in cells and TAG/TC assays indicated that 100 μM 5-HT significantly reduced approximately 50% of triglyceride and cholesterol content in the cells, decreasing the level of lipid droplet accumulation (Figures 2B–2D). Moreover, compared to the control group, treatment with 100 μM 5-HT also promoted an increase in intracellular calcium content (Figures 2E, 2F). It suppressed the mRNA expression of calcium efflux-related genes *PMCA1* and *SPCA1* while enhancing the mRNA expression of calcium influx-related genes *PMCA2* and *SPCA2* (Figure 2G).

### HTR2A overexpression elevated calcium levels and inhibited lipid droplet accumulation in goat mammary epithelial cells

**Figure 3:**
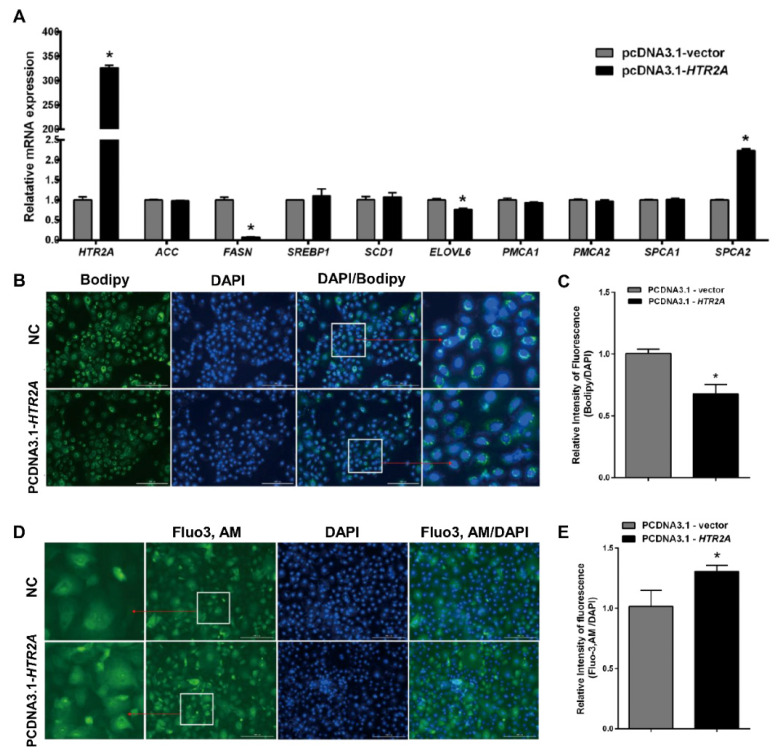
HTR2A overexpression elevated calcium levels and inhibited lipid droplet accumulation in GMECs. (A) RT-qPCR analysis of *ACC*, *FASN*, *SREBP1*, *SCD1*, *ELOVL6*, *PMCA1*, *PMCA2*, *SPCA1* and *SPCA2* expression in GMECs. (B,C) Bodipy staining and quantitative analysis of lipid droplets in GEMCs. (D,E) Fluo-3 staining and quantitative analysis of intracellular calcium in GMECs. GMECs were transfected with pcDNA3.1-*HTR2A* vector or empty vector. Scale bar, 200 μm. Error bars indicate means±SEM, *p≤0.05.DAPI, 4′,6-diamidino-2-phenylindole; NC, negative control; GMECs, goat mammary epithelial cells; RT-qPCR, real-time quantitative polymerase chain reaction; SEM, standard error of the mean.

To further confirm whether HTR2A is a key 5-HT receptor that regulates calcium and lipid metabolism in GMECs, HTR2A was overexpressed in GMECs. The expression of HTR2A in GMECs transfected with the overexpression vector significantly increased, concurrently decreasing the mRNA levels of *FASN* and *ELOVL6*, while promoting the mRNA expression of *SPCA2* (Figure 3A). Moreover, the overexpression of the HTR2A gene resulted in decreased lipid droplet accumulation in the cells (Figures 3B, 3C). Additionally, it was observed that the overexpression of the HTR2A gene also elevated the calcium level in the cells (Figures 3D, 3E).

### 5-HT inhibits triglyceride, total cholesterol, and lipid droplet accumulation in cells through HTR2A

**Figure 4:**
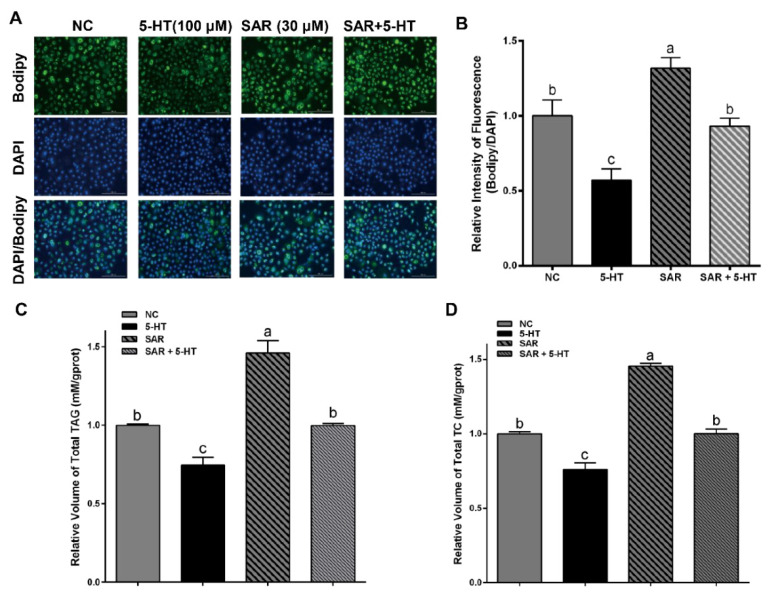
HTR2A mediated the Effect of serotonin on lipid genesis in GMECs. (A,B) Bodipy staining and quantitative analysis of lipid droplets in GEMCs. (C,D) TAG/TC analysis of concentrations of total TAG and TC in GMECs. GMECs were treated with 100 μM 5-HT and 30 μM SAR jointly or separately. Scale bar, 200 μm. Error bars indicate means±SEM, ^a–c^ Different letter denotes statistical defferences (p<0.05). NC, negative control; 5-HT, 5-hydroxytryptamine; DAPI, 4′,6-diamidino-2-phenylindole; SAR, sarpogrelate; TAG, triglycerides; TC, total cholesterol; GMECs, goat mammary epithelial cells; SEM, standard error of the mean.

To further confirm whether 5-HT exerts its regulatory function through the HTR2A receptor, we separately selected HTR2A-specific antagonist SAR to treat cells alone or in combination with 5-HT. Bodipy staining results showed that SAR treatment significantly increased lipid droplet content in GMECs. Additionally, triglyceride and total cholesterol test results indicated that SAR treatment significantly elevated levels of triglycerides and total cholesterol in GMECs. Compared to the SAR treatment alone group, the SAR+5-HT combined treatment group exhibited a significant decrease in triglyceride and total cholesterol levels. Compared to the control group, 5-HT treatment significantly reduced cellular lipid droplet levels, triglycerides, and total cholesterol content (Figure 4).

### The overexpression of HTR2A facilitates the activation of the AMPK/CaMKK**β** signaling pathway

**Figure 5:**
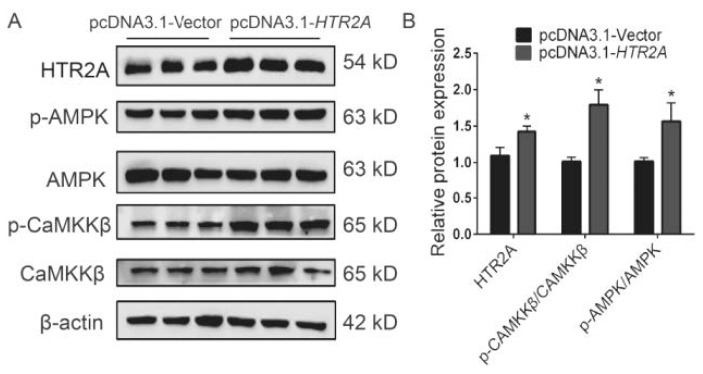
Effect of overexpression of HTR2A on the AMPK/CaMKKβ signaling pathway. (A) Western Blot analysis of the effect of HTR2A overexpression (pcDNA3.1-HTR2A) on the protein expression levels of HTR2A, AMPK, p-AMPK, CaMKKβ, and p-CaMKKβ. (B) Densitometry analysis of Western Blot analysis results. Error bars indicate means±SEM, * p≤0.05. SEM, standard error of the mean.

Finally, the experiment investigated the effect of HTR2A overexpression on the AMPK/CaMKKβ signaling pathway. Western blot analysis revealed that HTR2A overexpression significantly increased the expression level of HTR2A protein in GMECs, along with a significant rise in the levels of phosphorylated AMPK and phosphorylated CaMKKβ proteins (Figures 5A, 5B). This suggests that HTR2A overexpression may regulate lipid synthesis in GMECs by activating the AMPK/CaMKKβ signaling pathway.

## DISCUSSION

Peripheral 5-HT serves as a crucial regulator in the body [19], and in certain circumstances, its actions resemble those of hormone-like substances. Studies targeting various tissues such as the liver have reported 5-HT’s involvement in regulating lipid accumulation both in vivo and in vitro [19,20]. Although 5-HT has been recognized as a regulator of metabolic homeostasis in mammary tissue due to its involvement in regulating calcium metabolism, energy metabolism, and protein synthesis in mammary cells [21], its influence and function on lipid metabolism in the mammary gland remain unknown.

The RTCA technology employs a specialized process to integrate gold microelectrode sensor arrays at the bottom of each cell growth well in a cell culture plate. These sensors enable real-time, dynamic, and quantitative tracking of changes (values on the y-axis) in cell morphology and proliferation/differentiation [17]. When adherent cells growing on the microelectrode surface cause changes in the electrode interface impedance, these changes correlate with real-time alterations in the cell state. In this study, we observed that 5-HT (100 μM) induces an elevation in cellular calcium ion levels while concurrently reducing de novo synthesis and accumulation of lipid droplets in cells. The effects of HTR2A gene overexpression on cellular lipid and calcium metabolism were found closely resemble those of 5-HT. Furthermore, when cells were treated with receptor-specific antagonists, the regulatory effects of 5-HT and the influence of the HTR2A were both rescued. These findings suggest that HTR2A serves as a direct regulatory site for the dynamic regulation of mammary tissue lipid metabolism and calcium metabolism.

The effects of 5-HT on lipid metabolism appear to vary greatly among different animal organs and tissues. For example, in human or mouse studies, 5-HT induces excessive lipid accumulation and inflammatory lesions in the liver under high-fat conditions, which can be alleviated by selective TPH1 inhibitor or HTR2A gene knockdown [22]. However, Sumara et al. demonstrated that during fasting, intestinal-derived 5-HT increased the phosphorylation of hormone-sensitive lipase (HSL) and induced lipolysis through acting on HTR2B [23]. Under high-fat diet conditions, inhibiting peripheral 5-HT synthesis can improve mouse adiposity, reduce adipocyte size, and decrease mRNA levels of genes involved in lipogenesis [19].

For mammary tissue, its greatest functional difference from other organs lies in its ability to extract nutrients from the bloodstream and synthesize nutrients such as lipids and proteins within mammary epithelial cells, ultimately forming milk for excretion [24,25]. During the synthesis and secretion of milk, mammary epithelial cells must maintain a high-intensity, rhythmic, dynamic, and adaptive metabolic state [26]. Although lipid synthesis and calcium transport are two vastly different biological processes at the cellular level, they are indeed synchronized and correlated in mammary tissue [27]. In our previous studies, we primarily focused on 5-HT’s role in maintaining calcium metabolism homeostasis in mammary tissue. Through serendipitous experimentation, we discovered that 5-HT treatment of GMECs not only increased cellular calcium content but also significantly reduced lipid droplet accumulation, a phenomenon that had been overlooked in previous research. Therefore, we aims to comprehensively elucidate 5-HT’s regulatory role in lipid metabolism in goat mammary tissue.

Based on real-time cell viability analysis technology, a method relying on the rate of membrane potential growth within a unit of time to determine cell activity, it was found that the cell activity of GMECs exhibited dose-dependent growth, consistent with findings by Zhao et al [28], indicating that 5-HT promotes epithelial cell proliferation and inhibits apoptosis. Subsequently, through RT-PCR analysis of lipid synthesis-related gene expression in cells, it was observed that at a 5-HT concentration of 100 μM, there was a significant inhibition of de novo lipid synthesis in cells, which was confirmed by cell lipid droplet staining and measurements of triglyceride and cholesterol content. Concurrently, the calcium ion content in cells and the expression of calcium transport genes were regulated, consistent with previous reports [29]. Therefore, it can be inferred that in GMECs, 5-HT regulates lipid synthesis and also impacts calcium metabolism within the cells.

Currently, 10 types of 5-HT receptors have been identified in the mammary gland [30], providing evidence for the diversity of 5-HT functions in the mammary gland. It is possible that if specific receptors mediating 5-HT regulation of mammary lipid synthesis or calcium transport are identified, precise control of function could be achieved by targeting receptor expression or activity in animals. Previous research has confirmed that HTR7 is related to mammary gland development and milk protein synthesis [31], while HTR3 has been identified as a ligand-gated ion channel protein [32]. In this study, HTR2A was selected as the key receptor of interest because clues suggest that in liver and adipose tissue, HTR2A is associated with cellular lipid metabolism [14,15]. Additionally, HTR2A has been confirmed to interact with calcium-regulating proteins in cells [33]. The result provides evidence for the role of HTR2A in regulating lipid synthesis and calcium metabolism in GMECs from two perspectives. First, at the gene expression level, by constructing a HTR2A gene overexpression vector and transfecting cells, compared to the control group, the HTR2A overexpression group inhibited the expression of the key regulatory gene *FASN* involved in *de novo* synthesis and promoted the expression of *SPCA2* involved in calcium transport, reducing lipid accumulation and promoting an increase in calcium levels in cells, similar to the effect of 5-HT. Secondly, we selected SAR, a specific antagonist of HTR2A, to treat GMECs. The treatment concentration of 30 μM was determined as a low, non-cytotoxic concentration through the RTCA method (Supplement 3). The results demonstrated that when SAR was used to inhibit HTR2A activity in the cells, the level of lipid droplet accumulation significantly increased. The determination of the concentration of SAR used was primarily based on the results of pre-experimental screening for the optimal concentration.

Compared with the SAR-treated group, the 5-HT+SAR-treated group showed a significant decrease in lipid droplet levels in cells. Regarding this experimental phenomenon, the authors speculate that while HTR2A is a key receptor regulating cellular lipid synthesis, it is not the sole pathway for 5-HT to regulate intracellular lipid metabolism. A more straightforward proof is that overexpression of HTR2A does not alter the expression of genes except for *FASN*, *ELOVL6*, and *SPCA2*. Therefore, under the premise of limiting HTR2A activity, 5-HT can still reduce lipid droplet accumulation in cells, and the diversity of 5-HT-regulated cellular lipid metabolism pathways also provides the possibility for maintaining lipid homeostasis in cells. It is undeniable that there may be other non-5-HT-dependent pathways that can also affect the function of HTR2A and thereby regulate cellular function. However, the specific antagonistic effect of SAR on HTR2A is confirmed, and even the addition of SAR alone may produce a similar negative feedback regulatory effect. Recent studies have found that in addition to traditional receptor signaling pathways, 5-HT in organisms can also regulate cell function through epigenetic modifications such as 5-HT modification[34,35]. The de novo synthesis of lipids in mammary epithelial cells is primarily catalyzed by *CC* and *FASN* [18]. The Adenosine 5′-monophosphate (AMP)-activated protein kinase (AMPK) signaling pathway is the main pathway that negatively regulates cellular lipid synthesis. The main upstream activator of AMPK, CAMKKβ, is activated by changes in intracellular calcium levels following stimulation by certain hormones (such as adiponectin, leptin, etc.) [24,33]. In GMECs, although the specific molecular pathways by which 5-HT and HTR2A regulate GMECs lipid synthesis are not yet clear, current research results have proven that they promote the activation of the AMPK/CaMKKβ signaling pathway. Further in-depth studies on their molecular pathways are also necessary.

Therefore, further attention and effort are needed to elucidate the regulatory mechanisms of 5-HT and HTR2A on lipid synthesis and calcium metabolism in GMECs, which may help achieve the envisioned precise control of goat mammary gland lactation-related functions utilizing the characteristics of HTR2A.

## CONCLUSION

In summary, our findings elucidate the role of 5-HT in inhibiting lipid synthesis and accumulation while promoting an increase in calcium ion levels in mammary epithelial cells. Additionally, the specific receptor HTR2A has been identified to mediate the regulatory effects of 5-HT, with its specific antagonist capable of reversely promoting lipid droplet accumulation, triglyceride, and total cholesterol levels in cells. These results indicate that the expression or activity of 5-HT and its HTR2A receptor in goat mammary glands participate in regulating calcium metabolism and lipid metabolism homeostasis during lactation.

## SUPPLEMENTARY MATERIAL

**Supplement 1**. Immunofluorescence staining results of KRT18 protein in GMECs.

**Supplyment 2**. Primers for DNA cloning and qRT-PCR

**Supplyment 3**. The effects of 3 different concentration (shown by lines with different colors in the figure) of SAR within 24 hours on the activity of GMECs by RTCA assays.

## References

[1] Angeles-Agdeppa I, Capanzana MV, Li-Yu J, Schollum LM, Kruger MC (2010). High-calcium milk prevents overweight and obesity among postmenopausal women. Food Nutr Bull.

[2] Gomes JMG, de Assis Costa J, Ribeiro PVM, Alfenas RCG (2019). High calcium intake from fat-free milk, body composition and glycaemic control in adults with type 2 diabetes: a randomised crossover clinical trial. Br J Nutr.

[3] Suburu J, Shi L, Wu J (2014). Fatty acid synthase is required for mammary gland development and milk production during lactation. Am J Physiol Endocrinol Metab.

[4] Mamillapalli R, VanHouten J, Dann P (2013). Mammary-specific ablation of the calcium-sensing receptor during lactation alters maternal calcium metabolism, milk calcium transport, and neonatal calcium accrual. Endocrinology.

[5] Rapport MM, Green AA, Page IH (1948). Serum vasoconstrictor (serotonin): III. chemical inactivation. J Biol Chem.

[6] Berger M, Gray JA, Roth BL (2009). The expanded biology of serotonin. Annu Rev Med.

[7] Sahu A, Gopalakrishnan L, Gaur N (2018). The 5-hydroxytryptamine signaling map: an overview of serotonin-serotonin receptor mediated signaling network. J Cell Commun Signal.

[8] Matsuda M, Imaoka T, Vomachka AJ (2004). Serotonin regulates mammary gland development via an autocrine-paracrine loop. Dev Cell.

[9] Laporta J, Peters TL, Weaver SR, Merriman KE, Hernandez LL (2013). Feeding 5-hydroxy-l-tryptophan during the transition from pregnancy to lactation increases calcium mobilization from bone in rats. Domest Anim Endocrinol.

[10] Connelly MK, Cheng AA, Hernandez LL (2021). Graduate student literature review: serotonin and calcium metabolism: a story unfolding. J Dairy Sci.

[11] Zang WJ, Li H, Zhang ZF (2018). Serotonin induces parathyroid hormone-related protein in goat mammary gland. J Anim Sci.

[12] Zhang Z, Tian H, Chen X (2021). CRISPR/Cas9-mediated tryptophan hydroxylase 1 knockout decreases calcium transportation in goat mammary epithelial cells. Biochem Eng J.

[13] Laporta J, Moore SAE, Weaver SR (2015). Increasing serotonin concentrations alter calcium and energy metabolism in dairy cows. J Endocrinol.

[14] Choi W, Namkung J, Hwang I (2018). Serotonin signals through a gut-liver axis to regulate hepatic steatosis. Nat Commun.

[15] Yun J, Jin H, Cao Y (2018). RNA-Seq analysis reveals a positive role of HTR2A in adipogenesis in Yan yellow cattle. Int J Mol Sci.

[16] Shi H, Shi H, Luo J (2014). Establishment and characterization of a dairy goat mammary epithelial cell line with human telomerase (hT-MECs). Anim Sci J.

[17] Chen X, Zhang Z, Niu H (2024). Goat milk improves glucose metabolism in type 2 diabetic mice and protects pancreatic β-cell functions. Mol Nutr Food Res.

[18] Tian H, Luo J, Zhang Z (2018). CRISPR/Cas9-mediated stearoyl-CoA desaturase 1 (SCD1) deficiency affects fatty acid metabolism in goat mammary epithelial cells. J Agric Food Chem.

[19] Namkung J, Shong KE, Kim H, Oh CM, Park S, Kim H (2018). Inhibition of serotonin synthesis induces negative hepatic lipid balance. Diabetes Metab J.

[20] Crane JD, Palanivel R, Mottillo EP (2015). Inhibiting peripheral serotonin synthesis reduces obesity and metabolic dysfunction by promoting brown adipose tissue thermogenesis. Nat Med.

[21] Horseman ND, Collier RJ (2014). Serotonin: a local regulator in the mammary gland epithelium. Annu Rev Anim Biosci.

[22] Wang L, Fan X, Han J (2020). Gut-derived serotonin contributes to the progression of non-alcoholic steatohepatitis via the liver HTR2A/PPARgamma2 pathway. Front Pharmacol.

[23] Sumara G, Sumara O, Kim JK, Karsenty G (2012). Gut-derived serotonin is a multifunctional determinant to fasting adaptation. Cell Metab.

[24] Hansen HO, Tornehave D, Knudsen J (1986). Synthesis of milk specific fatty acids and proteins by dispersed goat mammary-gland epithelial cells. Biochem J.

[25] Li P, Fang X, Hao G (2023). Methionine promotes milk protein synthesis via the PI3K-mTOR signaling pathway in human mammary epithelial cells. Metabolites.

[26] Akersr MR, Bauman DE, Capuco AV, Goodman GT, Tucker AH (1981). Prolactin regulation of milk secretion and biochemical differentiation of mammary epithelial cells in periparturient cows. Endocrinology.

[27] Cameron CM, Rillema JA (1983). Extracellular calcium ion concentration required for prolactin to express its actions on casein, ribonucleic acid, and lipid biosynthesis in mouse mammary gland explants. Endocrinology.

[28] Zhao H, Chen S, Hu K (2021). 5-HTP decreases goat mammary epithelial cells apoptosis through MAPK/ERK/Bcl-3 pathway. Gene.

[29] Chen S, Zhao H, Yan X (2020). 5-Hydroxy-l-tryptophan promotes the milk calcium level via the miR-99a-3p/ATP2B1 axis in goat mammary epithelial cells. J Agric Food Chem.

[30] Suárez-Trujillo A, Argüello A, Rivero MA, Capote J, Castro N (2019). Short communication: differences in distribution of serotonin receptor subtypes in the mammary gland of sheep, goats, and cows during lactation and involution. J Dairy Sci.

[31] Pai VP, Hernandez LL, Stull MA, Horseman ND (2015). The type 7 serotonin receptor, 5-HT7, is essential in the mammary gland for regulation of mammary epithelial structure and function. BioMed Res Int.

[32] Fawley JA, Doyle MW, Andresen MC (2019). 5-HT3R–sourced calcium enhances glutamate release from a distinct vesicle pool. Brain Res.

[33] Turner JH, Raymond JR (2005). Interaction of calmodulin with the serotonin 5-hydroxytryptamine2A receptor: a putative regulator of G protein coupling and receptor phosphorylation by protein kinase C. J Biol Chem.

[34] Farrelly LA, Thompson RE, Zhao S (2019). Histone serotonylation is a permissive modification that enhances TFIID binding to H3K4me3. Nature.

[35] Al-Kachak A, Fulton SL, Di Salvo G (2023). Histone H3 serotonylation dynamics in dorsal raphe nucleus contribute to stress- and antidepressant-mediated gene expression and behavior. bioRxiv 539464 [Preprint].

