# Supplementary material for: Serotonin promotes calcium accumulation and inhibits lipid accumulation in cultured goat mammary epithelial cells through HTR2A
Source: Anim Biosci. 2025 Apr 4;38(8):1633–43. doi: 10.5713/ab.24.0792 (PMC12229898; doi:10.5713/ab.24.0792)
Supplement: Supplementary file 1 [file ab-24-0792-Supplementary-1.pdf]

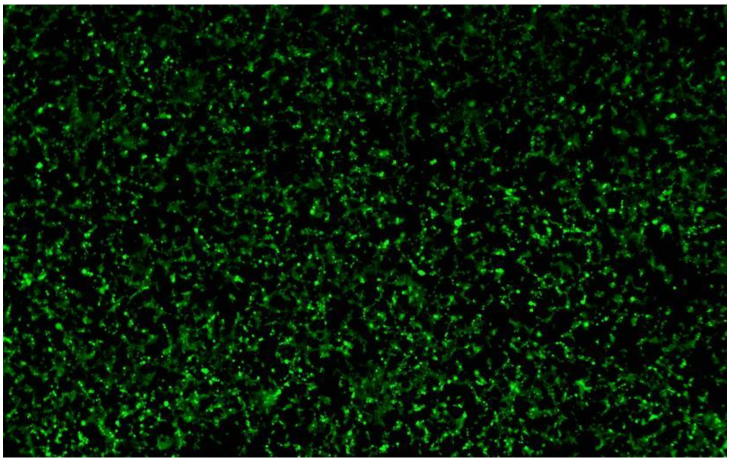

Supplement 1. Immunofluorescence staining results of KRT18 protein in GMECs.

Immunofluorescence staining for the identification of the Keratin 18 (KRT18) protein, a marker for mammary epithelial cells, was conducted in the cells. The results indicated expression of the KRT18 protein, confirming that the cells in use are indeed goat mammary epithelial cells. The green fluorescent portion represents the KRT18 protein.
