# Supplementary material for: Serotonin promotes calcium accumulation and inhibits lipid accumulation in cultured goat mammary epithelial cells through HTR2A
Source: Anim Biosci. 2025 Apr 4;38(8):1633–43. doi: 10.5713/ab.24.0792 (PMC12229898; doi:10.5713/ab.24.0792)
Supplement: Supplementary file 2 [file ab-24-0792-Supplementary-2.pdf]

Supplyment 2. Primers for DNA cloning and qRT-PCR

Gene name	GenBank accession number	Primer name	Primer sequence（5' to 3'）	Primer function
Serotonin receptor 2A (HTR2A)	XM_005687443.3	HTR2A-cds-F	ACATACGCCAGCCTCACT	Cloning of *HTR2A* CDS region
		HTR2A-cds-R	TTTCTCCAGTCTCCCAGT	
Serotonin receptor 2A (HTR2A)	XM_005687443.3	OE-*HTR2A*-F	*ATTTgcggccgc* ACATACGCCAGCCTCACT	Cloning of *HTR2A* for constructing pcDNA3.1-*HTR2A* vector
		OE-*HTR2A*-R	*CGggatcc* TTTCTCCAGTCTCCCAGT	
Serotonin receptor 2A (HTR2A)	XM_005687443.3	Flag-*HTR2A*-F	*CGggatccCG*ATGGATATTCTCTGTGAAGA	Cloning of *HTR2A* for constructing pEF-Neo-Flag-*HTR2A* vector
		Flag-*HTR2A*-R	*TTgcggccgcAA*TGGACACAGCTAACCT	
Serotonin receptor 2A (HTR2A)	XM_005687443.3	*HTR2A*-S188E-F	CGGTTCAACGAAAGAACTAAG	Cloning of *HTR2A* for constructing pEF-Neo-Flag-*HTR2A*-S188E vector
		HTR2A-S188E-R	CTTAGTTCTTTCGTTGAACCG	
Serotonin receptor 2A (HTR2A)	XM_005687443.3	*HTR2A*-S391E-F	TCGGCCTTTGAAAGGTATATT	Cloning of *HTR2A* for constructing pEF-Neo-Flag-*HTR2A*-S391E vector
		HTR2A-S391E-R	AATATACCTTTCAAAGGCCGA	
Serotonin receptor 2A (HTR2A)	XM_005687443.3	VN155-*HTR2A*-F	*ACGCgtcgac*ACATACGCCAGCCTCACT	Cloning of HTR2A for constructing p-BiFC-VN155-*HTR2A* vector
		VN155-*HTR2A*-R	*ATAAGAATgcggccgc*TTTCTCCAGTCTCCCAGT	
Serotonin receptor 2A (HTR2A)	XM_005687443.3	Si-*HTR2A*-F	GACGAUUCCAAAGUCUUUATT	siRNA for Si-*HTR2A*
		Si-*HTR2A*-R	UAAAGACUUUGGAAUCGUCTT	
Calmodulin (CaM)	XM_018062643.1	CaM-cds-F	CGGAGGAACCTTGATACCC	Cloning of *CaM* CDS region
		CaM-cds-R	CAGTCCATCAACTGGTGTTT	

Calmodulin (CaM)	XM_018062643.1	Myc-*CaM*-F<br>Myc-*CaM*-R	*CGggatccCG*ATGGCTGACCAGCTGACCGA<br>TTgcggccgcAATGCTTTGCAGTCATCATCT	Cloning of *CaM* for constructing pEF-Neo-Myc-*CaM* vector
Calmodulin (CaM)	XM_018062643.1	VC155-*CaM*-F<br>VC155-*CaM*-R	*CGgaattc*CGGAGGAACCTTGATACCC<br>*GGggtacc*CAGTCCATCAACTGGTGTTT	Cloning of *CaM* for constructing p-BiFC-VC155-*CaM* vector
Acetyl-CoA carboxylase 1 (ACC)	JN236219.1	q-*ACC*-F<br>q-*ACC*-R	CTCCAACCTCAACCACTACGG<br>GGGGAATCACAGAAGCAGCC	qRT-PCR for *ACC* gene
Fatty acid synthase (FASN)	DQ915966.3	q-*FASN*-F<br>q-*FASN*-R	GGGCTCCACCACCGTGTTCCA<br>GCTCTGCTGGGCCTGCAGCTG	qRT-PCR for *FASN* gene
Sterol regulatory element binding transcription factor 1 (SREBP1)	HM443643.1	q-*SREBP1*-F<br>q-*SREBP1*-R	CTGCTGACCGACATAGAAGACAT<br>GTAGGGCGGGTCAAACAGG	qRT-PCR for *SREBP1* gene
Stearoyl-CoA desaturase (SCD1)	GU947654	q-*SCD1*-F<br>q-*SCD1*-R	CCATCGCCTGTGGAGTCAC<br>GTCGGATAAATCTAGCGTAGCA	qRT-PCR for *SCD1* gene
ELOVL fatty acid elongase 6 (ELOVL6)	NM_001314257.1	q-*ELOVL6*-F<br>q-*ELONL6*-R	GGAAGCCTTTAGTGCTCTGGTC<br>ATTGTATCTCCTAGTTCGGGTGC	qRT-PCR for *ELOVL6* gene
Fatty acid binding protein 3 (FABP3)	NM_001285701.1	q-*FABP3*-F<br>q-*FABP3*-R	GATGAGACCACGGCAGATG<br>GTCAACTATTTCCCGCACAAG	qRT-PCR for *FABP3* gene
ATPase plasma membrane Ca^{2+} transporting 1 (PMCA1)	XM_018047613.1	q-*PMCA1*-F<br>q-*PMCA1*-R	ATTCTCACGGATGACAAT<br>CAACAATCACTGCTACTAC	qRT-PCR for *PMCA1* gene

ATPase plasma membrane Ca^{2+} transporting 2 (PMCA2)	XM_018038308.1	q-*PMCA2*-F<br>q-*PMCA2*-R	GCATTTTCATCGGGTTAGGAG<br>AGAGCTACGAAACGCCTTCAC	qRT-PCR for *PMCA2* gene
ATPase secretory pathway Ca^{2+} transporting 1 (SPCA1)	XM_018051409.1	q-*SPCA1*-F<br>q-*SPCA1*-R	GATACCTGTTCTGTGAGT<br>GATTAGACAATGGCTTCC	qRT-PCR for *SPCA1* gene
ATPase secretory pathway Ca^{2+} transporting 2 (SPCA2)	XM_005691840.3	q-*SPCA2*-F<br>q-*SPCA2*-R	GTGATTGGAACAGGAGAA<br>TGAGAAGAGTGTCAGTTG	qRT-PCR for *SPCA1* gene
Mechanistic target of rapamycin (mTOR)	XM_018059918.1	q-*mTOR*-F<br>q-*mTOR*-R	AAGCCGCGCGAACCTC<br>GGAGCTCCATGGTGACGTAG	qRT-PCR for *mTOR* gene
Serotonin receptor 2A (HTR2A)	XM_005687443.3	q-*HTR2A*-F<br>q-*HTR2A*-R	AGCTGATATGCTGCTGGGTT<br>GCCACCGGTACCCATAGAG	qRT-PCR for *HTR2A* gene
Ubiquitously expressed prefoldin like chaperone (UXT)	XM_005700842.2	q-UXT-F<br>q-UXT-R	TGTGGCCCTTGGATATGGTT<br>GGTTGTCGCTGAGCTCTGTG	qPCR for *UXT* gene<br>qPCR for *UXT* gene
Mitochondrial ribosomal protein L39 (MRPL39)	XM_005674737.3	q-MRPL39-F<br>q-MRPL39-R	TTGGTCAGAGCCCCAGAAGT<br>AGGTTCTCTTTTGTTGGCATCC	qPCR for *MRPL39* gene<br>qPCR for *MRPL39* gene
Ribosomal protein S9 (RPS9)	XM_018063497.1	q-RPS9-F<br>q-RPS9-R	CCTCGACCAAGAGCTGAAG<br>CCTCCAGACCTCACGTTTGTTC	qPCR for *RPS9* gene<br>qPCR for *RPS9* gene
