# Supplementary material for: Serotonin promotes calcium accumulation and inhibits lipid accumulation in cultured goat mammary epithelial cells through HTR2A
Source: Anim Biosci. 2025 Apr 4;38(8):1633–43. doi: 10.5713/ab.24.0792 (PMC12229898; doi:10.5713/ab.24.0792)
Supplement: Supplementary file 3 [file ab-24-0792-Supplementary-3.pdf]

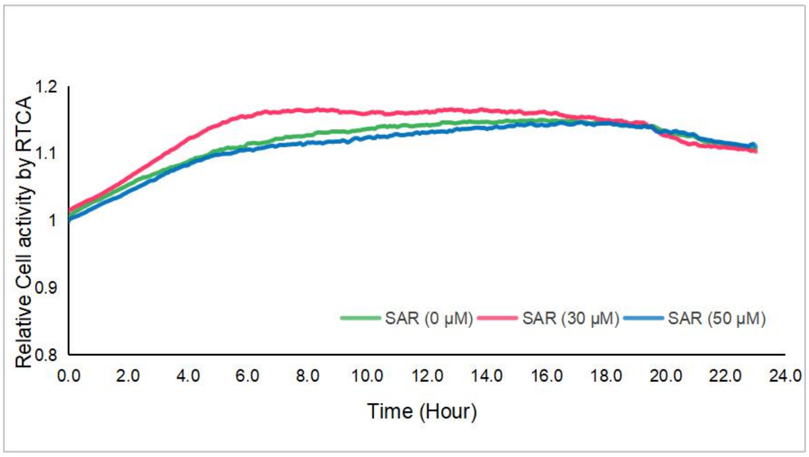

Supplyment 3. The effects of 3 different concentration (shown by lines with different colors in the figure) of SAR within 24 hours on the activity of GMECs by RTCA assays.

The determination of the concentration of SAR used was primarily based on the results of pre-experimental screening for the optimal concentration. After treating GMECs with different concentration gradients of 0 μM, 30 μM, and 50 μM, the method of real-time cell analysis (RTCA) assay was employed to detect changes in cell viability. The results indicated that at both 30 μM and 50 μM concentrations, SAR did not cause any damage to the cells (Figure R1). Consequently, for the formal experiments, the following experiments chose the lower concentration of 30 μM SAR for cell treatment.
